# The putative *Escherichia coli* dehydrogenase YjhC metabolises two dehydrated forms of N-acetylneuraminate produced by some sialidases

**DOI:** 10.1042/BSR20200927

**Published:** 2020-06-24

**Authors:** Takfarinas Kentache, Léopold Thabault, Alessio Peracchi, Raphaël Frédérick, Guido T. Bommer, Emile Van Schaftingen

**Affiliations:** 1 Laboratory of Physiological Chemistry, de Duve Institute, UCLouvain, Brussels, B-1200, Belgium; 2 Medicinal Chemistry Research Group (CMFA), Louvain Drug Research Institute (LDRI), UCLouvain, Brussels, B-1200, Belgium; 3 Department of Chemistry, Life Sciences and Environmental Sustainability, University of Parma, Parma 43124, Italy; 4 Walloon Excellence in Life Sciences and Biotechnology (WELBIO), UCLouvain, Brussels, B-1200, Belgium

**Keywords:** dehydratase, dehydrogenase, Escherichia coli, sialic acid

## Abstract

Homologues of the putative dehydrogenase YjhC are found in operons involved in the metabolism of N-acetylneuraminate (Neu5Ac) or related compounds. We observed that purified recombinant YjhC forms Neu5Ac from two dehydrated forms of this compound, 2,7-anhydro-N-acetylneuraminate (2,7-AN) and 2-deoxy-2,3-didehydro-N-acetylneuraminate (2,3-EN) that are produced during the degradation of sialoconjugates by some sialidases. The conversion of 2,7-AN into Neu5Ac is reversible and reaches its equilibrium when the ratio of 2,7-AN to Neu5Ac is ≈1/6. The conversion of 2,3-EN is irreversible, leading to a mixture of Neu5Ac and 2,7-AN. NMR analysis of the reaction catalysed by YjhC on 2,3-EN indicated that Neu5Ac was produced as the α-anomer. All conversions require NAD^+^ as a cofactor, which is regenerated in the reaction. They appear to involve the formation of keto (presumably 4-keto) intermediates of 2,7-AN, 2,3-EN and Neu5Ac, which were detected by liquid chromatography-mass spectrometry (LC-MS). The proposed reaction mechanism is reminiscent of the one catalysed by family 4 β-glycosidases, which also use NAD^+^ as a cofactor. Both 2,7-AN and 2,3-EN support the growth of *Escherichia coli* provided the repressor NanR, which negatively controls the expression of the *yjhBC* operons, has been inactivated. Inactivation of either YjhC or YjhB in NanR-deficient cells prevents the growth on 2,7-AN and 2,3-EN. This confirms the role of YjhC in 2,7-AN and 2,3-EN metabolism and indicates that transport of 2,7-AN and 2,3-EN is carried out by YjhB, which is homologous to the Neu5Ac transporter NanT.

## Introduction

N-acetylneuraminate (Neu5Ac), the most abundant form of sialic acid, is an anionic 9-carbon keto sugar abundantly present in animals, where it serves to decorate secreted proteins, and membrane proteins and lipids. It is found at the extremities of mature forms of N-glycans as well as of some O-glycans, and in glycolipids such as gangliosides. It is modified in various manners, mainly by acetylation or methylation of hydroxyl groups, and in animals but not in humans, by hydroxylation of its N-acetyl group to an N-glycolyl group. Sialic acids are particularly abundant at the cell surface and in the mucin layer of epithelia. Many bacteria that live in close association with animals are able to metabolise Neu5Ac. Some of them use it for decorating their glycolipids or for producing polysialic acids-based capsules [1–3].

The metabolism of Neu5Ac involves its release from sialoconjugates by sialidases. These enzymes, which act in the outer space of Enterobacteriaceae and of other bacteria such as *Streptococcus*, release Neu5Ac as its α-anomer, but some of them also release dehydrated forms of this molecule, either 2,7-anhydro-N-acetylneuraminate (2,7-AN) or 2-deoxy-2,3-didehydro-N-acetylneuraminate (2,3-EN) [4–6]. Further metabolism of Neu5Ac has been particularly well studied in *Escherichia coli* (which does not possess sialidases, and therefore takes advantage of the Neu5Ac that is released from sialoconjugates by other bacteria). Transport of Neu5Ac involves its crossing the outer membrane by the general porins OmpC/OmpF and by NanC [7], and its transport by the permease NanT across the inner membrane [8].

Once in the cytosol, Neu5Ac will be cleaved by an aldolase reaction to pyruvate and N-acetylmannosamine. This reaction shows preference for the α-anomer of Neu5Ac. N-acetylmannosamine is then phosphorylated by NanK and epimerised to N-acetylglucosamine 6-phosphate, which can be deacetylated and deaminated to the glycolytic intermediate, fructose-6-phosphate. This metabolic pathway allows Neu5Ac to support growth of *E. coli*.

The present work was initiated to identify the function of YjhC, a putative dehydrogenase encoded by operons involved in Neu5Ac metabolism and whose role was then completely unknown. Its presence in Neu5Ac operons is intriguing because there is no obvious need for a dehydrogenase in Neu5Ac metabolism. As explained in the present paper, we found that YjhC is involved in the metabolism of two dehydrated forms of Neu5Ac that are produced by streptococcal sialidases B and C. *E. coli* can grow on these two forms of Neu5Ac and they are transported by YjhB, a putative transporter encoded by the same operon as YjhC.

While this work was in progress, two articles appeared on the function of the same or an orthologous protein. Horn and co-workers reported the crystal structure of *E. coli* YjhC and showed that it displays a weak dehydrogenase activity on Neu5Ac, suggesting that this compound could be a physiological substrate for this enzyme [9]. Bell and co-workers showed that YjhC from *Ruminococcus gnavus*, which shares 61 percent sequence identity with *E. coli* YjhC, serves to metabolise 2,7-AN to Neu5Ac, allowing this dehydrated form of sialic acid to be used as a substrate for the bacterium [10].

The purpose of the present paper is to report our findings on the metabolic function of YjhC and to propose a reaction mechanism that accounts for the need of a reversible dehydrogenation step.

## Experimental

### Bacterial strains

The clones encoding the YjhC, the Neu5Ac lyase NanA and the Neu5Ac mutarotase NanM [11] were from the ASKA library, which comprises a complete set of ORF clones of *E. coli* K-12 strain [12]. ORFs are cloned in the pCA24N vector in which His6-tag is located at the N-terminal side of the multiple cloning site. Of note, pCA24N confers resistance to chloramphenicol [12]. The genes encoding (i) *Neisseria meningitidis* CMP-sialic acid synthetase (*Nmcss*), (ii) *Pasteurella multocida* sialyltransferase 1 M144D mutant (*Pmst1_M144D*) [13] and (iii) *Streptococcus pneumoniae* TIGR4 sialidase B (*SpnanB*) were purchased from Integrated DNA Technologies (Leuven, Belgium). All primers used in this work were also from Integrated DNA Technologies.

Strains *E. coli* K12 *∆nanR::kan, ∆yjhB::kan* and *∆yjhC::kan* were obtained from the Keio collection [14]. The kanamycin resistance cassettes were excised using pCP20 [15] to generate *∆nanR* mutant. Double mutants *∆nanR-∆yjhB* and *∆nanR-∆yjhC* were generated in the *∆nanR* strain by P1 transduction procedure [16] using alleles from *∆yjhB::kan* and *∆yjhC::kan* strains followed by *kanR* removal using pCP20. The pAM238 vector was used to rescue *yjhB* and *yjhC* in *∆nanR-∆yjhB* and *∆nanR-∆yjhC* strains, respectively. DNA sequences were verified by DNA sequencing performed by Genewiz (Leipzig, Germany).

### Preparation of *Nmcss, Pmst1_M144D* and *SpnanB* in pET22b(+) vector

*Nmcss, Pmst1_M144D* and *SpnanB* were PCR-amplified by using the DNA polymerase Phusion® (Thermo Scientific) according to the manufacturer’s recommendations. The primers used for *Nmcss* were: forward primer 5′-AACAACATATGGAAAAACAAAATATTGC-3′ (NdeI restriction site is underlined) and reverse primer CGCCTCGAGGCTTTCCTTGTGATTATG-3′ (XhoI). For *Pmst1_M144D*: forward primer was GAGTCCATATGAAAACAATCACGCTGTATTTAG-3′ (NdeI) and 5′-TTGTCCTTTCTCGAGCAACTGTTTTAAACTG-3′ (XhoI) for the reverse one. The forward primer of *SpnanB* was 5′-GATCGGATCCGAATGAATTAAACTATGGTCAACT-3′ (BamHI) and reverse primer was 5′-CGCCTCGAGTTTTGTTAAATCATTAATTTCCAAA-3′ (XhoI). The resulting PCR products were purified and digested with their respective restriction enzymes (all from Thermo Scientific). The purified and digested PCR product was ligated with pre-digested pET22b(+) vector and transformed into chemically competent *E. coli* XL1 Blue cells. Selected clones were grown to prepare minipreps and DNA sequences were confirmed by DNA sequencing. The recombinant vectors were then transformed into chemically competent strain *E. coli* BL21 (DE3) for protein expression.

### Expression and purification of recombinant enzymes

For NanA, YjhC, NmCSS, PmST1_M144D and sialidase B expression, *E. coli* BL21 recombinant strains were cultured in LB medium (10 g/l tryptone, 5 g/l yeast extract and 10 g/l NaCl) (Sigma–Aldrich, U.S.A.) supplemented with ampicillin at 200 µg/ml (chloramphenicol at 25 µg/ml for NanA and YjhC). Expression of the enzymes was achieved by induction with 0.1 mM of isopropyl-1-thio-β-d-galactopyranoside (IPTG) when OD_600 nm_ reached 0.4–0.6. This was followed by incubation at 37°C (20°C for sialidase B expression) for 24 h with shaking at 200 rpm in an INFORS HT Multitron Standard incubator shaker (INFORS AG, Switzerland). For NanM expression, the strain was cultured in M9 minimal medium (Millipore, U.S.A.) supplemented with 0.2% glucose, 1 mM MgCl_2_, 0.1 mM CaCl_2_, 0.1% Casamino acids, 25 µg/ml chloramphenicol and 0.5 µg/ml, biotin and thiamine. Once induced by IPTG, the bacterial culture was incubated at 20°C for 24 h.

The cells were harvested by centrifugation at 6000 ***g*** for 20 min and the pellet was resuspended (50 ml/l cell culture) in lysis buffer (50 mM HEPES, pH 7.5, 1 mM DTT, 2 µg/ml antipain, 2 µg/ml leupeptin, 0.5 mM phenylmethylsulfonyl fluoride (PMSF), 1 mg/ml lysozyme, 0.3 M NaCl). The mixture was freeze-thawed for two cycles in liquid nitrogen. The lysate was treated with DNase I (0.2 mg/ml and 5 mM MgCl_2_) for 60 min on ice, then centrifuged at 16000 ***g*** for 50 min and the supernatant was collected.

All His6-tagged proteins were purified from the clarified lysate using an AKTA purifier 900 series (GE Healthcare) with a Ni^2+^-resin column (HisTrap™-HP 1 ml, GE Healthcare). The mobile phase consisted of equilibration buffer (10 mM imidazole, 0.3 M NaCl, 30 mM HEPES pH 7.5, 2 µg/ml antipain/leupeptin) and the elution buffer (500 mM imidazole, 0.3 M NaCl, 30 mM HEPES, pH 7.5, 2 µg/ml antipain/leupeptin). Retained proteins were eluted at a flow rate of 1 ml/min, using a one-step linear gradient from 0 to 100% of elution buffer over 30 min. The 1-ml fractions containing the purified enzymes were pooled and concentrated to 2.5 ml by using VivaSpin 15 centrifugal filter units according to the manufacturer’s recommendations (Sartorius Stedim, Goettingen, Germany). After that, desalting was performed by using PD-10 Sephadex™ G-25 M columns (GE Healthcare, U.K.). Proteins were collected in elution buffer (30 mM HEPES pH 7.5, 300 mM KCl,1 mM DTT, 2 µg/ml antipain/leupeptin). Glycerol was then added to purified proteins at 10% and aliquots were stored at −80°C for further analyses.

Purity of the recombinant proteins was estimated by SDS/PAGE and Coomassie Blue staining. SDS/PAGE was performed in commercial NuPage™ 12% Bis-Tris-glycine gels (Invitrogen, Germany) using a Novex Mini-Cell gel electrophoresis unit (Invitrogen, China) at 180 V and 170 mA. PageRuler™ plus Prestained Protein Ladder 10–180 kDa (Thermo Scientific, Lithuania) were used as molecular weight standards. Gels were stained with Coomassie Blue R-250.

Protein concentration in the purified preparations was estimated by measuring A_280_ and computing the concentration from the expected extinction coefficient (42900 M^−1^.cm^−1^ for YjhC) on the basis of the amino acid composition (Protparam tool, at https://web.expasy.org/protparam/). For YjhC we took into account the contribution of NAD^+^ and NADH to the absorbance at 280 nm.

### Synthesis of 2,7-anhydro-Neu5Ac

2,7-AN was synthesised as previously reported [17]. The synthesis procedure was stopped by adjusting the pH above 12 with NaOH and heating to 100°C for 10 min to destroy residual Neu5Ac. Strong anion exchange chromatography (SAX) was performed using Dowex® 1×8:200–400 mesh (Acros Organics, NJ, U.S.A.) resin in a 20 cm × 1.5 cm column. Gel filtration chromatography was performed with a column (50 cm × 1 cm) packed with Bio-Gel P-2 Fine resin (Bio-Rad). The peristaltic pump P-1 and the fractionator LKB RediFrac (both from Pharmacia) were used for both SAX and gel filtration chromatographies with a flow rate of 1 ml/min. 2,3-EN was purchased from Carbosynth, U.K.

### Measurement of Neu5Ac dehydratase activity

The reaction mixture contained 30 mM HEPES, pH 7.5, 10 mM KCl, 1 mM DTT, 1 mM MgCl_2_ and 0.5 mg/ml bovine serum albumin. The mixture was supplemented with Neu5Ac (Carbosynth, U.K.) at 5 mM. YjhC was added at 1.4 µM in a final volume of 1 ml. After incubation at 37°C for 2 h, the reaction was then stopped by (i) quenching for liquid chromatography-mass spectrometry (LC-MS) analysis (see below in ‘LC-MS analyses’ section) or (ii) heating to 80°C for 5 min for enzymatic assay or gas chromatography-mass spectrometry (GC-MS) analyses.

### Measurement of the concentrations of Neu5Ac and its anhydro forms by enzymatic assay

The measurement was performed spectrophotometrically at 340 nm in an assay mixture containing 30 mM HEPES buffer, pH 7.5, 10 mM KCl, 1 mM DTT, 1 mM MgCl_2_, 0.5 mg/ml bovine serum albumin, 10 µM NAD^+^ and 150 µM NADH at 37°C. The Neu5Ac lyase NanA (0.3 µM), Neu5Ac mutarotase NanM (0.1 µM), and lactate dehydrogenase (0.8 µM) (LDH; Roche, Germany) were first added to exhaust Neu5Ac. YjhC (0.45 µM) was then added to convert 2,7-AN and/or 2,3-EN into Neu5Ac. The change in NADH concentration was used to calculate the concentration of Neu5Ac and (2,7-AN + 2,3-EN).

### Effect of NAD^+^ concentration on YjhC activity

YjhC activity was assessed *in vitro* in an assay mixture containing 30 mM HEPES buffer, pH 7.5, 10 mM KCl, 1 mM DTT, 1 mM MgCl_2_, 0.5 mg/ml bovine serum albumin with 0.45 µM YjhC and 1 mM substrate (2,7-AN or 2,3-EN), as well as variable concentrations of NAD^+^. The assay was performed in a volume of 200 µl and at 37°C. After incubation (15 min when the substrate was 2,7-AN and 45 min for 2,3-EN), the reactions were arrested by heating at 80°C for 5 min. The concentration of residual substrates and of formed Neu5Ac were measured spectrophotometrically with a Beckman Coulter DU-800 series machine.

### Effect of divalent cations

The impact of divalent cations on YjhC activity was assessed. The tested concentrations were 50 μM for Mg^2+^, Mn^2+^, Cd^2+^, Co^2+^, Ca^2+^, or Ni^2+^ and 5 μM for Zn^2+^ and Cu^2+^. Ethylene glycol-bis-2-aminoethylether (EGTA, Sigma–Aldrich) was tested at 5 mM. The reaction buffer contained 30 mM HEPES, pH 7.5, 10 mM KCl, 1 mM DTT, 1 mM MgCl_2_, 0.5 mg/ml bovine serum albumin, and 50 μM NAD^+^, but DTT was omitted when Cu^2+^ was added. The mixture contained 150 μM of 2,7-AN and 0.3 µM of YjhC. The coupling enzymes were NanA (1.6 µM), NanM (1.2 µM) and LDH (2.4 µM). YjhC activity was monitored spectrophotometrically.

### Effect of pH on the 2,7-AN hydratase activity of YjhC

The effect of pH was assessed with a two-step assay (as in Figure 3). The assay mixture contained the following buffers at 50 mM: MES (pH 5.5–6.5), HEPES (pH 7–8) or Tris (8.5–9), as well as 10 mM KCl, 1 mM DTT, 1 mM MgCl_2_, 50 µM NAD^+^, 0.5 mg/ml bovine serum albumin, 0.45 µM YjhC and 1 mM 2,7-AN. The assay was performed in a volume of 200 µl and at 37°C. After 15-min incubation, the reactions were arrested by heating at 80°C for 5 min. Concentrations of residual 2,7-AN and formed Neu5Ac were measured spectrophotometrically as described in section ‘Measurement of the concentrations of Neu5Ac and the anhydro forms by enzymatic assay’.

### Kinetic analyses

Kinetic parameters of YjhC were determined at 37°C in an assay mixture containing, unless otherwise stated, 30 mM HEPES buffer, pH 7.5, 10 mM KCl, 1 mM DTT, 1 mM MgCl_2_, 0.5 mg/ml bovine serum albumin, 50 µM NAD^+^ using 0.3 µM YjhC. The following 2,3-EN or 2,7-AN concentrations were tested: 25, 50, 75, 100, 125, 150, 175, 200, 225, 250, 275, 300, 325, 500, 1000, 2000, and 4000 µM. The dependence of the observed rate on substrate concentration was analysed using Prism 8.1.3 (GraphPad, CA, U.S.A.) based on an allosteric sigmoidal equation.

### GC-MS and LC-MS analyses

All GC-MS analyses were performed as reported previously [18]. For LC-MS analyses, 20 µl of enzymatic assay mixtures were quenched with 180 µl of cold methanol-chloroform (9:1 v/v). The resulting mixture was vigorously shaken for 5 min at 4°C, incubated on ice for 15 min and then centrifuged at 16000 ***g*** for 15 min; 100 µl of supernatant were then loaded in vials for LC-MS injection. All LC-MS experiments were performed on a 1290 HPLC system coupled to an ESI-QTOF iFunnel 6550 series MS (both from Agilent Technologies). In initial experiments the chromatographic separation was performed using an Inertsil 3-μm particle ODS-4 column (150 × 2.1 mm; GL Biosciences) under conditions described previously [19]. Later experiments were analysed on a smaller ODS-4 column (20 × 2.1 mm; GL Biosciences) at a constant flow rate of 0.2 ml/min with mobile phase A consisting of 5 mM hexylamine (Sigma–Aldrich, U.S.A.) adjusted to pH 6.3 with acetic acid (Biosolve BV, The Netherlands) and phase B consisting of 90% methanol (Biosolve BV, The Netherlands)/10% of 10 mM ammonium acetate (Biosolve BV, The Netherlands) adjusted to pH 8.5 with ammonia (Merck, Darmstadt, Germany). The injection volume was 5 μl. The mobile phase profile consisted of the following steps and linear gradients: 0–3 min at 0% B; 3–6 min from 0 to 5% B; 6–8 min from 5 to 10% B; 8 –12 min at 10% B; 12–13 min from 10 to 40% B; 13–15 min from 40 to 100% B; 15–16 min at 100% B; 16–17 min at 0% B. The mass spectrometer was operated in negative mode using an electrospray ionisation source under the following conditions: ESI spray voltage 3500 V, sheath gas 350°C at 11 l/min, nebuliser pressure 35 psig and drying gas 200°C at 14 l/min. A range of *m/z* 69–1150 was scanned by combining 8131 transients leading to a 1-s cycle time. Compounds were identified based on their exact mass and retention time compared with standards. Extracted-ion chromatograms of the [M-H]-forms were integrated using Mass Hunter software (Agilent, CA, U.S.A.). LC-MS results were obtained from at least three independent experiments.

### NMR analysis

All experiments were performed on a Bruker Ascend Avance III 600 MHz system equipped with a broadband cryoprobe (Bruker). Experiments at pH 6 were conducted at 10 or 20°C in 10% D_2_O containing 50 mM sodium deuterated acetate buffer, 10 mM NaCl and 100 µM of 3-(trimethylsilyl)propionic 2,2,3,3-d4 acid sodium salt (TMSP) as a chemical shift reference (0 ppm). Experiments at pH 7 were conducted at 10°C in 10% D_2_O containing 20 mM sodium phosphate buffer, 10 mM NaCl and 100 µM TMSP as a chemical shift reference (0 ppm). Water signal suppression was achieved using an excitation-sculpting scheme. Thirty-two scans were collected for each spectrum to yield a 15K points-free induction decay.

## Results

### YjhC converts dehydrated forms of Neu5Ac into Neu5Ac

**Figure 1:**
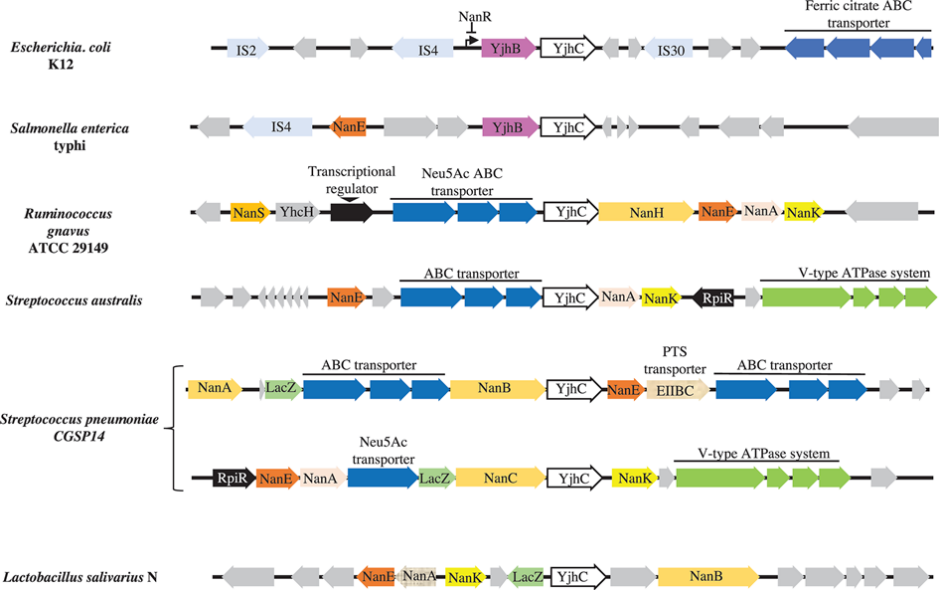
Organisation of genomic regions harbouring *yjhC* in *E. coli* K12, *Salmonella enterica Typhi, R. gnavus* ATCC 29149, *Streptococcus australis, S. pneumoniae* CGSP14, and *Lactobacillus salivarius* LacZ: β-galactosidase; NanA in pink arrow: N-acetylneuraminate pyruvate lyase; NanA in beige arrow: sialidase A; NanB: sialidase B; NanC: sialidase C; NanE: N-acetylmannosamine-6-P epimerase; NanH: sialidase H; NanK: N-acetylmannosamine kinase; NanS: N-acetylneuraminate esterase; RpiR: Neu5Ac utilisation regulator; YhcH: putative epimerase; YjhB: putative permease; IS: Transposase.

Figure 1 shows the genomic environment of YjhC in several bacterial genomes. YjhC homologues are present in many bacteria in the vicinity of a (putative) Neu5Ac transporter and of enzymes of Neu5Ac metabolism such as sialidase (NanB), Neu5Ac aldolase (NanA), N-acetylmannosamine kinase (NanK) and N-acetylmannosamine-6-phosphate epimerase (NanE). These findings, and the fact that the *yjhC* gene is up-regulated by Neu5Ac [20] led us to test the possibility that YjhC is a dehydrogenase acting on Neu5Ac.

**Figure 2:**
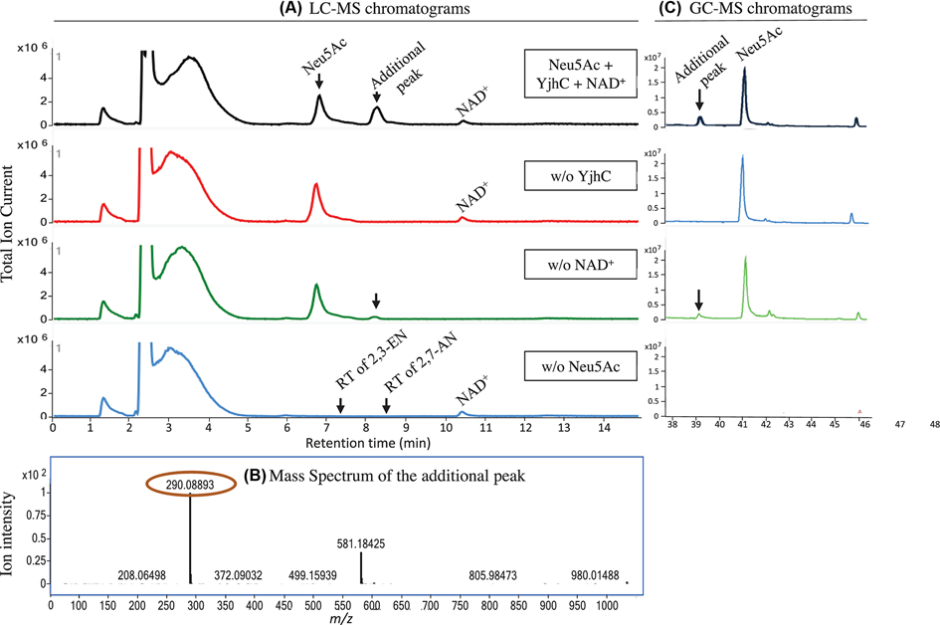
YjhC forms 2,7-AN from Neu5Ac LC-MS (**A**) and GC-MS (**C**) profiles showing the formation of an anhydro form of Neu5Ac (*m/z* = 290.08814, **B**) upon incubation of 1 mM Neu5Ac with YjhC (1.49 µM), with or without 30 µM NAD^+^. This anhydro form coelutes with 2,7-AN and not with the 2,3-EN. One representative experiment is shown.

To investigate the function of *E. coli* YjhC, we produced the recombinant protein and we incubated it with or without Neu5Ac and NAD^+^. Analysis by LC-MS of the incubation mixture revealed the formation of a new peak at 8 min in the samples containing Neu5Ac. This peak was stronger in the incubations containing NAD^+^ (Figure 2A). Analysis of the peak MS spectrum indicated the presence of a major ion with *m/z* 290.0725 (Figure 2B), consistent with a dehydrated form of Neu5Ac. This indicated that a 2,3-ene form of Neu5Ac or a 2,7-dehydration product might be formed. When compared with standards, the peak appearing in the incubation mixtures eluted at the same time as 2,7-AN, but not 2,3-EN*.* Analysis of the reaction mixtures by GC-MS after trimethylsilylation demonstrated the appearance of a peak (Figure 2C) with the same MS spectrum as 2,7-AN (Supplementary Figure S1) confirming that YjhC is able to convert Neu5Ac into the 2,7-dehydrated form.

**Figure 3:**
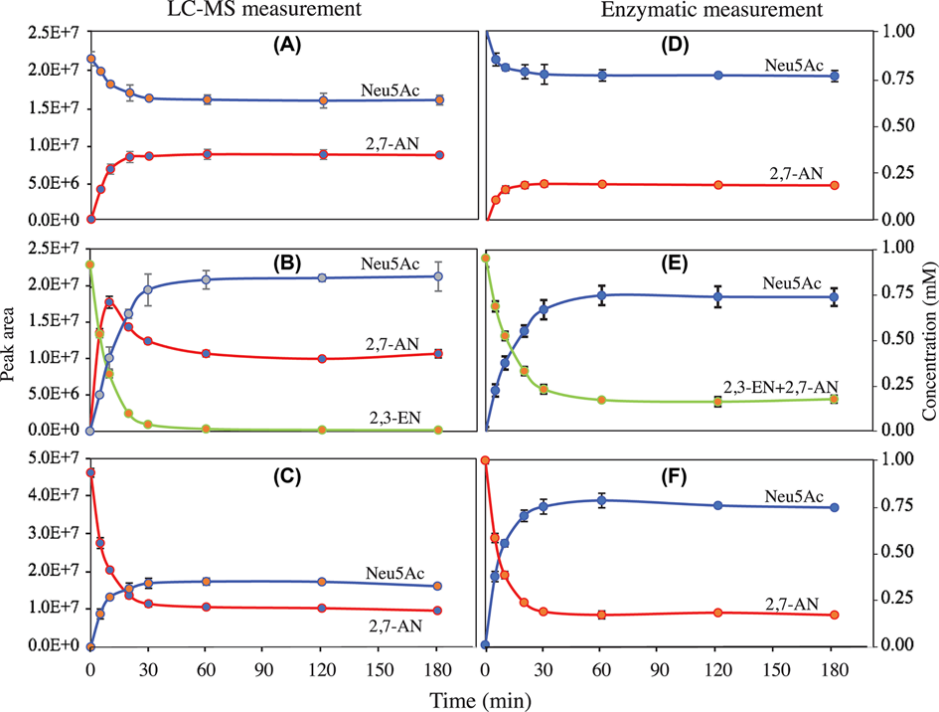
Conversion of Neu5Ac, 2,3-EN and 2,7-AN by YjhC Purified YjhC (0.45 µM) was incubated with 1 mM Neu5Ac (**A,D**), 2,3-EN (**B,E**) or 2,7-AN (**C,F**) for the indicated times at 37°C. The reactions were arrested by chloroform-methanol extraction (A–C) or by heating (D–F). Metabolites were quantified by LC-MS analysis (A–C) or by spectrophotometric assay (D–F). Values are means ± SD from three experiments.

We explored further the specificity of YjhC by testing its ability to metabolise Neu5Ac, 2,3-EN and 2,7-AN in the presence of NAD^+^. LC-MS analysis of the reaction products indicated that Neu5Ac was converted into 2,7-AN (Figure 3A) and that reciprocally 2,7-AN was converted into Neu5Ac (Figure 3C). Both reactions did not reach completion, indicating that the thermodynamic equilibrium was attained. YjhC also acted on 2,3-EN, converting it into a mixture of Neu5Ac and 2,7-AN until 2,3-EN was completely exhausted (Figure 3B). Remarkably, the 2,7-AN concentration went through a transient maximum after 10 min before going down to approximately 70% of this transient maximum. LC-MS measurement did not allow us to determine quantitatively the extent of the reaction, because different substrates generate different ionic currents. To analyse in a quantitative manner the equilibrium reached at the end of the reactions, we used a spectrophotometric assay allowing the measurement of Neu5Ac with NanA and the sum of 2,7-AN and 2,3-EN with YjhC (see ‘Experimental’ section). This approach allowed us to conclude that the reaction reached its equilibrium when the ratio of Neu5Ac on 2,7-AN was approximately 6 to 1 at 37°C (Figure 3D–F).

### Requirement for NAD^+^ and other potential cofactors

**Figure 4:**
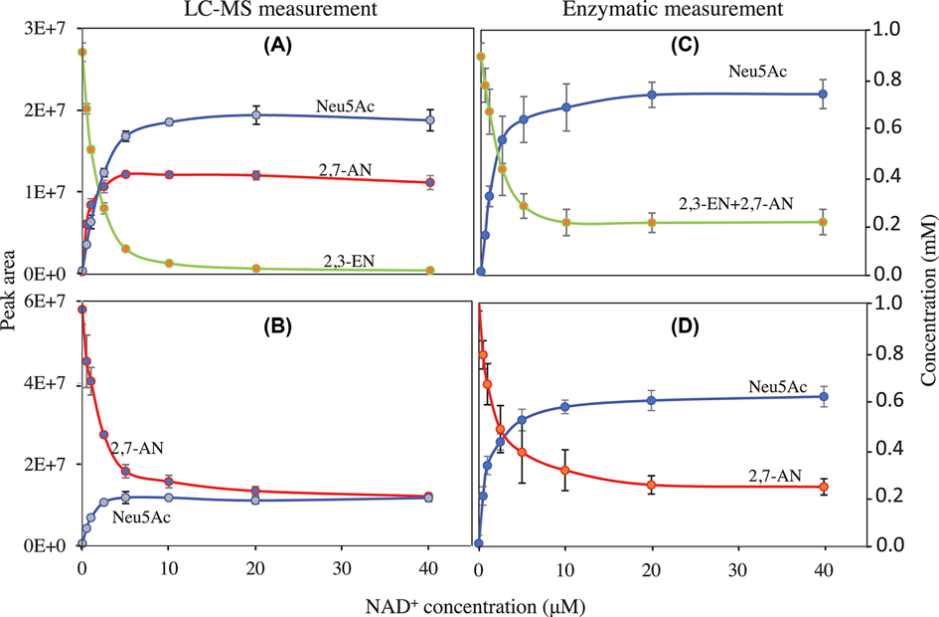
Effect of NAD^+^ on enzymatic activity Effect of NAD^+^ concentration on the conversion of 2,3-EN (**A,C**) and 2,7-AN (**B,D**). NAD^+^ dependency of YjhC activity was monitored *in vitro* in an assay mixture containing 30 mM HEPES buffer, pH 7.5, 10 mM KCl, 1 mM DTT, 1 mM MgCl_2_, 0.5 mg/ml bovine serum albumin with 0.45 µM YjhC and 1 mM substrate (2,7-AN or 2,3-EN), as well as variable concentrations of NAD^+^. The assay was performed in a volume of 200 µl and at 37°C. After incubation (20 min when the substrate was 2,7-AN and 60 min for 2,3-EN), the reactions were arrested by chloroform-methanol extraction (A,B) for LC-MS assays or by heating at 80°C for 5 min (for enzymatic assays, C,D). Values are means ± SD from three experiments.

Figure 4 shows the effect of NAD^+^ concentration on the conversion of 2,3-EN and of 2,7-AN into Neu5Ac. In both cases, the formation of Neu5Ac was strongly stimulated by NAD^+^, though there was a detectable activity in its absence, corresponding to about ≈3% of the maximal activity. This effect was not observed with NADP^+^, NADH or NADPH (not shown). The calculated *K*_a_ was 1.01 and 0.84 µM for the conversion of 2,3-EN and 2,7-AN, respectively.

As noted, the enzyme showed a detectable activity (≈3 percent of the Vmax) in the absence of added NAD^+^. This suggested that the enzyme preparation contained some of this cofactor. LC-MS analysis of the preparation purified from bacteria by metal affinity chromatography and gel filtration indicated the presence of small amounts of NAD^+^ and NADH (≈0.094 and 0.008 mole/mole of enzyme subunit, respectively), while the preparations of other bacterial enzymes (*E. coli* NanA and NanM, *S. pneumoniae* sialidase B, which are not dehydrogenases) showed no detectable or much lower levels of NAD^+^ and no detectable NADH (Supplementary Figure S2).

**Figure 5:**
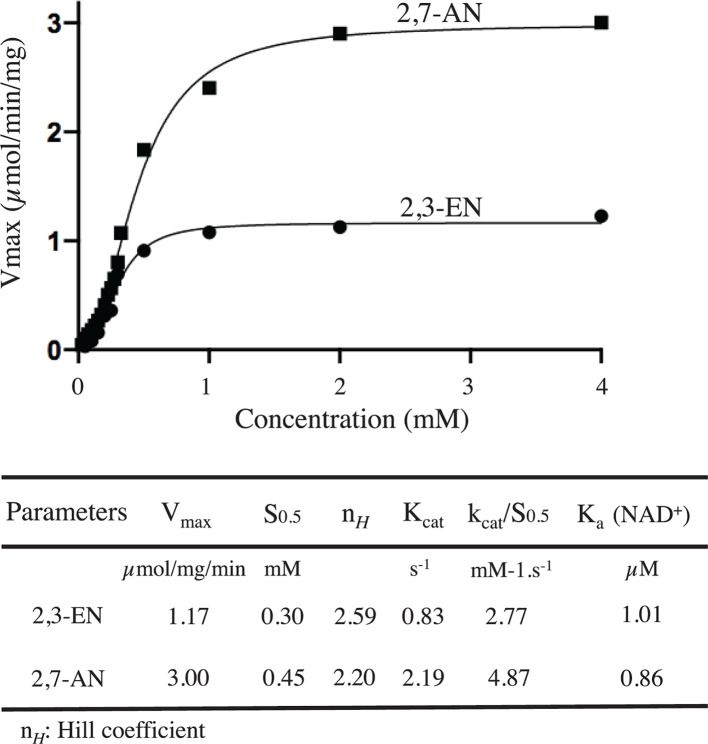
Saturation curve of YjhC for 2,7-AN and 2,3-EN The activity was assayed spectrophotometrically in an assay mixture containing 30 mM HEPES buffer, pH 7.5, 10 mM KCl, 1 mM DTT, 1 mM MgCl_2_, 0.5 mg/ml bovine serum albumin, 50 µM NAD^+^ and 150 µM NADH using 0.3 µg/ml YjhC and the indicated concentrations of 2,3-EN or 2,7-AN. The coupling enzymes were NanA (1.6 µM), NanM (1.2 µM) and LDH (2.4 µM). Kinetic data parameters obtained by fitting the data to an allosteric sigmoidal equation (using Prism 8; GraphPad, CA, U.S.A.) are shown in the table.

Determination of the kinetic properties of YjhC in the presence of a saturating concentration of NAD^+^ (50 µM) yielded sigmoidal titration for both 2,7-AN and 2,3-EN, with Hill coefficients of 2.20 and 2.59, S_0.5_ of 0.45 and 0.30 mM and *V*_max_ of ≈3.0 and 1.17 µmol/min/mg protein (Figure 5). The presence of EGTA (5 mM) and several divalent cations (MgCl_2_, MnCl_2_, CdCl_2_, CoCl_2_, CaCl_2_, NiCl_2_, all at 50 µM; ZnCl_2_ and CuCl_2_ at 5 µM) did not affect the activity. The pH dependence activity showed an optimum between pH 7.5 and 8 (Supplementary Figure S3).

### Evidence for keto forms as reaction intermediates

**Figure 6:**
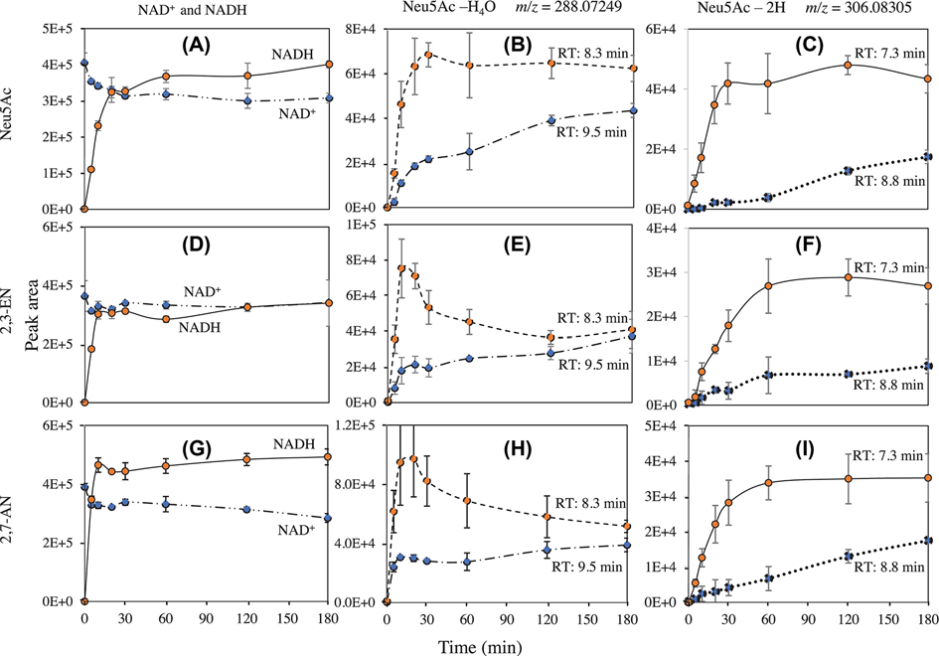
Time course of the formation of NADH and reaction intermediates from Neu5Ac, 2,3-EN and 2,7-AN YjhC (0.45 µM) was incubated for the indicated times in the presence of 1 mM Neu5Ac (**A–C**), 2,3-EN (**D**–**F**) or 2,7-AN (**G–I**) and 30 µM NAD^+^. Same experiment as in Figure 3 and Supplementary Figure S4. The intermediates are designated by their rounded *m/z* values and their retention time.

The fact that NAD^+^ stimulated the reaction suggested that the reaction mechanism involved the transient formation of oxidised forms of Neu5Ac and of its dehydrated forms as intermediates in the reaction, as well as of NADH. We noted indeed, that in the presence of NAD^+^ and 2,3-EN, 2,7-AN or Neu5Ac, YjhC catalysed the formation of small amounts of NADH (up to ≈3 µM) (Figure 6). We noted also the formation of two dehydrogenated forms of Neu5Ac (*m/z* 306.08305), eluting at 7.3 and 8.8 min (called 306/7.3 and 306/8.8), which we hypothesised to be 4-keto-Neu5Ac and 3,4-ene-Neu5Ac, respectively, and two forms of dehydrogenated, dehydrated Neu5Ac (*m/z* 288.07249) eluting at 8.3 and 9.5 min (288/8.3 and 288/9.5) (Supplementary Figure S4). The latter two correspond most likely to keto forms of 2,3-EN and 2,7-AN, respectively. Since unmodified 2,3-EN elutes before 2,7-AN, it is likely that the species eluting at 8.3 min is keto-2,3-EN and the one eluting at 9.5 min is keto-2,7-AN. Figure 6 shows the time course of the formation of these species from Neu5Ac, 2,3-EN and 2,7-AN (same experiments as in Figure 3). Of note, the ratio of the putative keto-2,3-EN/keto-2,7-AN was, at short times, somewhat higher when 2,3-EN was used as a substrate (the ratios were 4.36 and 4.19 at 5 and 10 min, respectively; Figure 6E) than when 2,7-AN was used as a substrate (2.58 and 3.09, Figure 6H), which is consistent with the structure assignments (see ‘Discussion’ section). In addition, these ratios were 6.23 and 4.25 when Neu5Ac was used as substrate (Figure 6B). The effect of the NAD^+^ concentration on the formation of NADH and of these intermediates is further illustrated in Supplementary Figure S5.

### NMR analysis shows that YjhC produces the α-anomer of Neu5Ac

**Figure 7:**
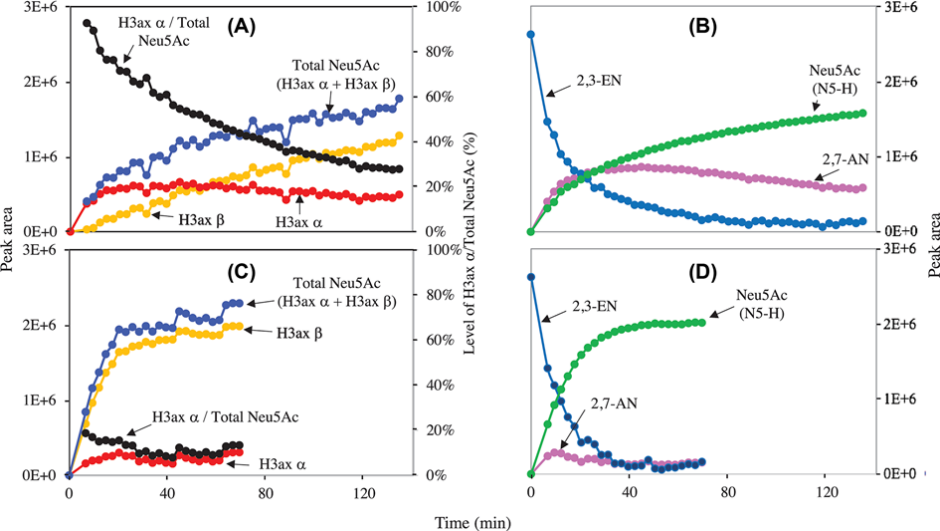
YjhC produces the α-anomer of Neu5Ac from 2,3-EN 2,3-EN (1 mM) was incubated at 10°C in 50 mM deuterated acetate buffer, pH 6 in the presence of 20 µM NAD^+^, 3 µM YjhC, without (**A,B**) or with (**C,D**) and 0.8 µM NanM. Repetitive NMR spectra (32 scans, 2.5 min) were taken at different times after initiation of the reaction by addition of YjhC. The α and β forms of NeuAc (A,C) were quantified from the shifts of axial H3 at 1.65 and 1.8 ppm. The total amount of Neu5Ac shown in (A,C) and the proportion of α form were computed from these values. The amount of 2,3-EN, 2,7-AN and Neu5ac shown in (B,D) were calculated from the shifts of N5-H in the 8 ppm region (see spectra in Supplementary Figure S5).

To determine which anomeric form of Neu5Ac is produced from 2,3-EN, we analysed the formation of YjhC products by NMR under conditions where spontaneous anomerisation of Neu5Ac was slowed down (i.e., at low temperature and acidic pH) [11]. The shifts that correspond to the two H3 are clearly distinct in the α form of Neu5Ac (≈2.8 and 1.65 ppm for H3eq and H3ax, respectively) compared with the β form (≈2.2 and 1.8 ppm, respectively) (Supplementary Figure S6) and they were used to quantify the two anomeric forms. As shown in Figure 7A,B, 2,3-EN was initially converted into the α-anomer of Neu5Ac, while the β-anomer appeared more slowly with time. At the first time at which an NMR spectrum was recorded (≈6.7 min), the proportion of α-anomer represented ∼92% of the formed Neu5Ac. This proportion progressively decreased with time in agreement with a slow anomerisation rate. The same incubation performed in the presence of Neu5Ac anomerase [11] resulted, as expected, in the accumulation of much lower amounts of the α-anomer and much higher amounts of the β-anomer, which rapidly reached the expected equilibrium (Figure 7C,D). Interestingly, the formation of 2,7-AN was much higher in the absence of anomerase than in its presence, presumably due to the lower concentration of the α-anomer of Neu5Ac in the latter condition. As expected, lower proportions of α-anomer were observed initially when the NMR spectra were recorded (at 6.7 min) on samples incubated at a higher temperature (45% at 20°C and pH 6) or a higher pH (33% at 10°C and pH 7).

### YjhC and YjhB are required for the metabolism of 2,3-EN and 2,7-AN

Since YjhC is able to convert 2,3-EN and 2,7-AN into Neu5Ac, we anticipated that these two Neu5Ac derivatives would be able to support the growth of *E. coli*. Trials made with wild-type *E. coli* K12 using a minimal medium with different potential energetic substrates confirmed that *E. coli* grows on Neu5Ac (Supplementary Figure S7B). In contrast, cells failed to grow on 2,3-EN or 2,7-AN (Supplementary Figure S8C,D). However, we noted that growth was more rapid in the presence of a mixture of 5 mM 2,3-EN and 5 mM Neu5Ac, than in the presence of 5 mM Neu5Ac alone (not shown). Furthermore, when cultured in the presence of both 2,3-EN and Neu5Ac, 2,3-EN was consumed in the medium, while this was not the case in cultures where only 2,3-EN was present (and no growth occurred). As the operon encoding YjhB and YjhC is under the control of the repressor NanR [20], these findings suggested that 2,3-EN, unlike Neu5Ac, is unable to relieve the repression exerted by NanR on operons involved in Neu5Ac metabolism. This interpretation was confirmed by the finding that a *∆nanR* mutant grew on 2,3-EN and 2,7-AN (Supplementary Figure S7C,D).

Next, we inactivated either *yjhC* or *yjhB* in the *∆nanR* background. This revealed that *yjhC* and *yjhB* genes are both required for the growth on 2,3-EN and 2,7-EN, indicating that not only YjhC, but also the putative permease YjhB is indispensable for the growth on these two compounds (Supplementary Figure S7C,D). To discard the possibility that the *∆yjhB* mutant acted by a polar effect that would prevent the expression of functional YjhC, we tested spectrophotometrically the YjhC activity in bacterial extracts from different mutants grown on LB medium. As expected, we found that the ‘2,3-ene hydratase activity’ of the *∆nanR-∆yjhB* double mutant was comparable with that of the *∆nanR* single mutant, while that of the *∆nanR-∆yjhC* mutant was nil. In agreement with the regulation of the *yjhBC* operon by NanR, we also noted that the YjhC activity was nil in extracts from wild-type *E. coli* grown on LB medium.

## Discussion

*E. coli* YjhC, a putative dehydrogenase, has long been thought to be involved in the metabolism of Neu5Ac or of some closely related molecule. The main message of the present paper is that YjhC serves to metabolise two ‘dehydrated’ compounds, 2,7-AN and 2,3-EN, which are made from sialoconjugates by sialidases B and C [4], respectively. This work confirms and extends therefore recently published work showing that the *R. gnavus* YjhC homologue, which shares ∼61% sequence identity with *E. coli* YjhC, serves to hydrolyse 2,7-AN to Neu5Ac [10]. We also confirm that the latter reaction is reversible. In addition, we demonstrate that YjhC catalyses the irreversible hydration of 2,3-EN, leading to the formation of a mixture of Neu5Ac and 2,7-AN in a ≈6 to 1 ratio. Of note, YjhC is the first enzyme reported to metabolise efficiently 2,3-EN. *Streprococcus* sialidase C, which converts sialoconjugates into 2,3-EN, slowly hydrates this compound to Neu5Ac, but the reaction takes 24 h to be completed [4].

**Figure 8:**
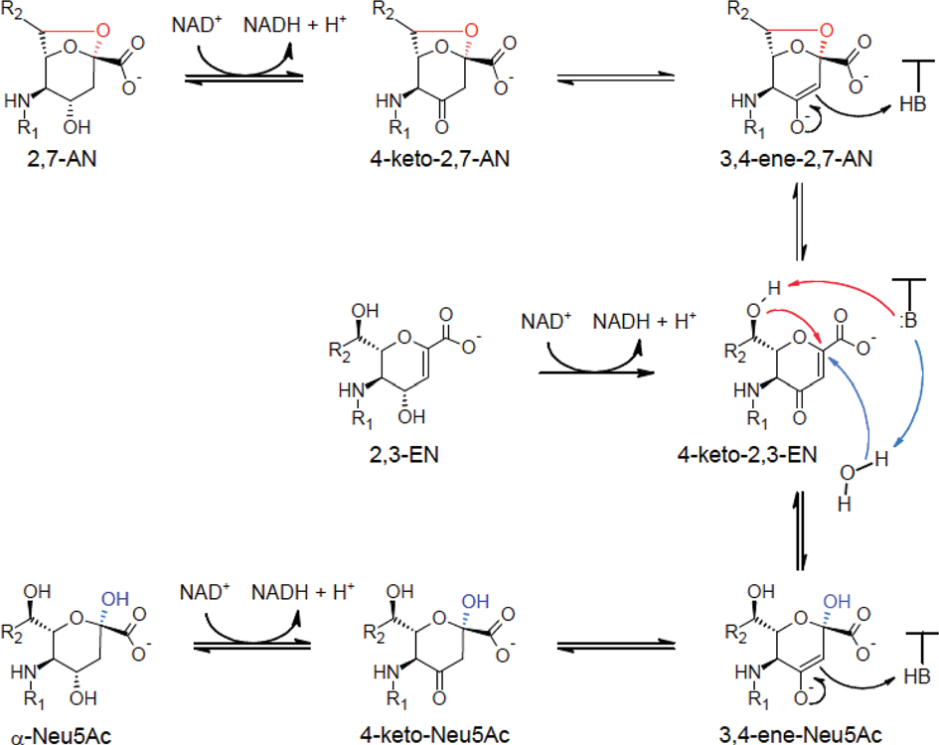
Tentative mechanism for the conversion of 2,3-EN and 2,7-AN into Neu5Ac The mechanism that we propose involves three keto forms (of Neu5Ac, 2,3-EN and 2,7-AN), and the enolates corresponding to 4-keto-Neu5Ac and keto-2,7-AN. The latter are formed by attack of C2 of 4-keto-2,3-EN by OH- in the first case and by O- bound to C7 in the second case. We assume that the enolates are stabilised by an unidentified positive charge in the catalytic site of YjhC. Protonation of their C3 leads to 4-keto-Neu5Ac and 4-keto-2,7-AN, which are then reduced in the enzyme to Neu5Ac and 2,7-AN, respectively. We tentatively identified four of the five intermediates (see text). We suggest that the enol form of 4-keto-Neu5Ac is stabilised by an internal hydrogen bond formed with the oxygen of the acetyl group bound to N5 and that this is why it is observable in free form.

YjhC belongs to the Gfo/Idh/MocA dehydrogenase family [9], which comprises glucose-fructose oxidoreductase, inositol-2-dehydrogenase, and many other enzymes acting most often on a pyranose structure [21]. In a recent paper reporting the structure of YjhC, it was shown that this protein exerted a weak dehydrogenase activity on Neu5Ac [9]. Substrate docking modelling suggested that the dehydrogenation took place on carbon 4 of the Neu5Ac molecule [9]. We show in the present work that NAD^+^ is indispensable for the interconversions of Neu5Ac, 2,3-EN and 2,7-AN to proceed and we tentatively propose a reaction mechanism that involves 4-keto intermediates (Figure 8). According to this model, the overall hydration reaction that this enzyme catalyses on 2,3-EN would start by dehydrogenation to a 4-keto intermediate, which would then undergo an attack on C2 by a hydroxyde ion to produce an enolate. The latter would be protonated on C3, to yield 4-keto-Neu5Ac, and finally reduced to Neu5Ac by the NADH present in the catalytic site. Conversion of 2,7-AN into Neu5Ac would involve its oxidation to 4-keto-2,7-AN and an internal elimination reaction leading to opening of the 2,7-bond and formation of 4-keto-2,3-EN. Subsequent reactions are as described above for the hydration of 2,3-EN.

The reaction mechanism that we suggest is similar to the one that has been proposed for the hydrolysis of glycoside by glycosidases of the type 4 family [22,23]. These enzymes also belong to a dehydrogenase family (different from that of YjhC) and catalyse an overall hydrolysis reaction that involves a dehydrogenation reaction, an elimination reaction, a water addition reaction and, finally, reduction of the keto group by the NADH generated in the catalytic site during the first step. A main difference is that the reaction mechanism of glycosidases 4 involves the utilisation of a divalent cation (Mn^2+^), which presumably stabilises an intermediate enolate, while in the present case, there is no evidence for the requirement of a divalent cation.

In support of the mechanism that we propose, LC-MS analysis provided evidence for the formation of four intermediates. Two of them have *m/z* values corresponding to dehydrogenated 2,3-EN or 2,7-AN. We tentatively identify them as 4-keto-2,3-EN and 4-keto-2,7-AN for the first and second species that elute from the column, in agreement with the order of elution of the parent compounds, 2,3-EN and 2,7-AN, but also with the observation that the ratio of the putative 4-keto-2,3-EN/4-keto-2,7-AN is, at the initial times of the reaction, slightly higher when 2,3-EN is metabolised than when 2,7-AN is metabolised. This difference in the ratio of intermediates is consistent with the expected predominant flux from 4-keto-2,3-EN to 4-keto-2,7-AN when 2,3-EN is metabolised and conversely when 2,7-AN is metabolised. The location of the keto function on carbon 4 is consistent with the structure of YjhC, and also with the reaction mechanism that is proposed, as in this position, the keto group facilitates the formation of a conjugated double bond between C2 and C3.

We tentatively identify the two dehydrogenated forms of Neu5Ac as 4-keto-Neu5Ac and 3,4-ene-Neu5Ac for the first and second eluting species. This is consistent with their ratio at the initial times of the reaction proceeding from Neu5Ac on the one hand or from 2,3-EN or 2,7-AN on the other hand. Part of the oxidised intermediates that we observed most likely leaked out of the catalytic site, as indicated by the fact that the concentration of the formed NADH exceeded the concentration of enzyme by a factor of ∼5. We speculate nonetheless that most of the catalytic cycles occur without detachment of NADH and of the intermediates.

In this mechanism, hydration of 4-keto-2,3-EN and addition of O7 to form 4-keto-2,7-AN are expected to proceed from the same side of the double bond. As 2,7-AN is in the α configuration, it is expected that the hydration reaction of 4-keto-2,3-EN to form Neu5Ac leads also to the formation of the α-anomer. This was supported by the NMR analysis of the reaction product, which showed that a large proportion of Neu5Ac was in the α configuration at beginning of NMR analysis.

The reaction mechanism that we propose indicates the presence of common intermediates for the hydrolysis of 2,7-AN and 2,3-EN. This is not only supported by the findings of the same four intermediates but also by the finding that during the conversion of 2,3-EN into Neu5Ac and 2,7-AN, the accumulation of 2,7-AN is strongly enhanced if the anomerisation of the α-anomer to the β-anomer is limited (see Figure 7).

Finally, the reaction converting 2,3-EN into a mixture of Neu5Ac and 2,7-AN is irreversible. This irreversibility appears to occur at the level of the oxidation of 2,3-EN to 4-keto-2,3-EN, since the putative 4-keto-2,3-EN is observed. This is not surprising as this reaction leads to the formation of a pair of conjugated double bonds and is therefore expected to be more exergonic than the similar oxidation reactions occurring with Neu5Ac and 2,7-AN.

The substrate saturation curves for 2,3-EN and 2,7-AN are sigmoidal. This could indicate that the enzyme has allosteric properties. The Hill coefficients are, however, higher than the subunit number. This suggests that other explanations for the sigmoid behaviour [24] may be also involved. Further studies are needed to elucidate this point and to understand the physiological advantage that such a kinetic behaviour may confer.

**Figure 9:**
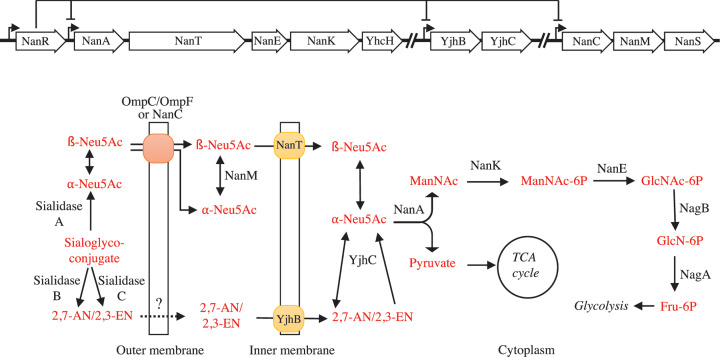
Assimilation and catabolic pathway of Neu5Ac, 2,3-EN and 2,7-AN in *E. coli* K12 Metabolites are designated by red and proteins by black lettering. Most of these proteins are encoded in three gene clusters: *nanATEK-yhcH, yjhBC* and *nanCMS*. Expression of these clusters are regulated by the repressor NanR located immediately upstream of *nanA.* Of note, sialidases are not present in *E. coli.*

The operon *yjhBC* is under the control of the repressor NanR (Figure 9) [20]. To demonstrate the involvement of YjhC and YjhB in the metabolism of 2,3-EN and 2,7-AN, we had to use NanR mutants, because neither 2,3-EN, nor 2,7-AN are apparently able to cause derepression, which is required for the expression of genes involved in sialic acid utilisation. Studies with mutants created in NanR-deficient strains indicated that both YjhC and YjhB are required for the growth on 2,3-EN and 2,7-AN. This not only supports the *in vitro* data on the enzymatic function of YjhC, but also indicates that the putative transporter of unknown function YjhB, serves to transport both 2,3-EN and 2,7-AN and that these two molecules are not transported by NanT.

Search of homologues indicates the presence of homologues of YjhC in many genomes. As expected, it is often found close to genes encoding enzymes involved in Neu5Ac metabolism and most interestingly sialidases. Remarkably, in *Streptoccus*, two homologues of YjhC are found, one close to the sialidase B and the other close to the sialidase C gene, i.e*.* the enzymes that produce 2,7-AN and 2,3-EN, respectively. Putative transporters are often associated with these operons. Most of them belong to the ABC transporters family. In the case of *R. gnavus* the associated ABC transporter RUMGNA_02698 was shown to allow the transport of 2,7-AN [10]. In *E. coli* and in closely related species, the transport is carried out by YjhB, which belongs to the proton symporter family. Thus at least two very different types of transporters are involved in the metabolism of 2,7-AN (and most likely 2,3-EN) in different organisms.

The identification of the role of the putative dehydrogenase YjhC in the metabolism of the anhydro sugar 2,7-AN may also shed light on the metabolism of another anhydro sugar, levoglucosan (1,6-anhydro-β-D-glucopyranoside), which in *Arthrobacter* can be converted into 3-keto-levoglucosan [25] by levoglucosan dehydrogenase [25]. This reaction is likely followed by hydrolysis of the anhydro bond and reduction of the resulting 3-keto-glucose to glucose [25]. The three reactions allowing the conversion of levoglucosan into glucose would be similar to those allowing the conversion of 2,7-AN into Neu5Ac by YjhC. As levoglucosan dehydrogenase is homologous to YjhC [26], one has to consider the possibility that the three reactions be catalysed by one single enzyme.

In conclusion, our work extends previous findings made on *R. gnavus* and *E. coli* YjhC [9,10] by showing that the *E. coli* enzyme has not only the ability of metabolising 2,7-AN, but also 2,3-EN; that the weak Neu5Ac dehydrogenase activity described by Horne et al. [9] participates in a more global reaction leading to hydration of 2,3-EN and 2,7-AN and that the conversion of 2,7-AN into Neu5Ac proceeds with retention of configuration. Furthermore, YjhC is functionally associated with the transporter YjhB, which utilises the same substrates and allows both 2,3-EN and 2,7-AN to serve as energetic substrates for *E. coli*.

## Supplementary Material

Supplementary Figures S1-S8

## Abbreviations

2,3-EN: 2-deoxy-2,3-didehydro-N-acetylneuraminate
2,7-AN: 2,7-anhydro-N-acetylneuraminate
EGTA: ethylene glycol-bis-2-aminoethylether-N,N,N′,N′-tetraacetic acid
GC-MS: gas chromatography-mass spectrometry
IPTG: isopropyl-1-thio-β-d-galactopyranoside
LC-MS: liquid chromatography-mass spectrometry
LDH: lactate dehydrogenase
Neu5Ac: N-acetylneuraminate
TMSP: 3-(trimethylsilyl)propionic 2,2,3,3-d4 acid sodium salt
