# Supplementary material for: The putative Escherichia coli dehydrogenase YjhC metabolises two dehydrated forms of N-acetylneuraminate produced by some sialidases
Source: Biosci Rep. 2020 Jun 24;40(6):BSR20200927. doi: 10.1042/BSR20200927 (PMC7315737; doi:10.1042/BSR20200927)
Supplement: Supplementary Figures S1-S8 [file BSR-2020-0927_supp.pdf]

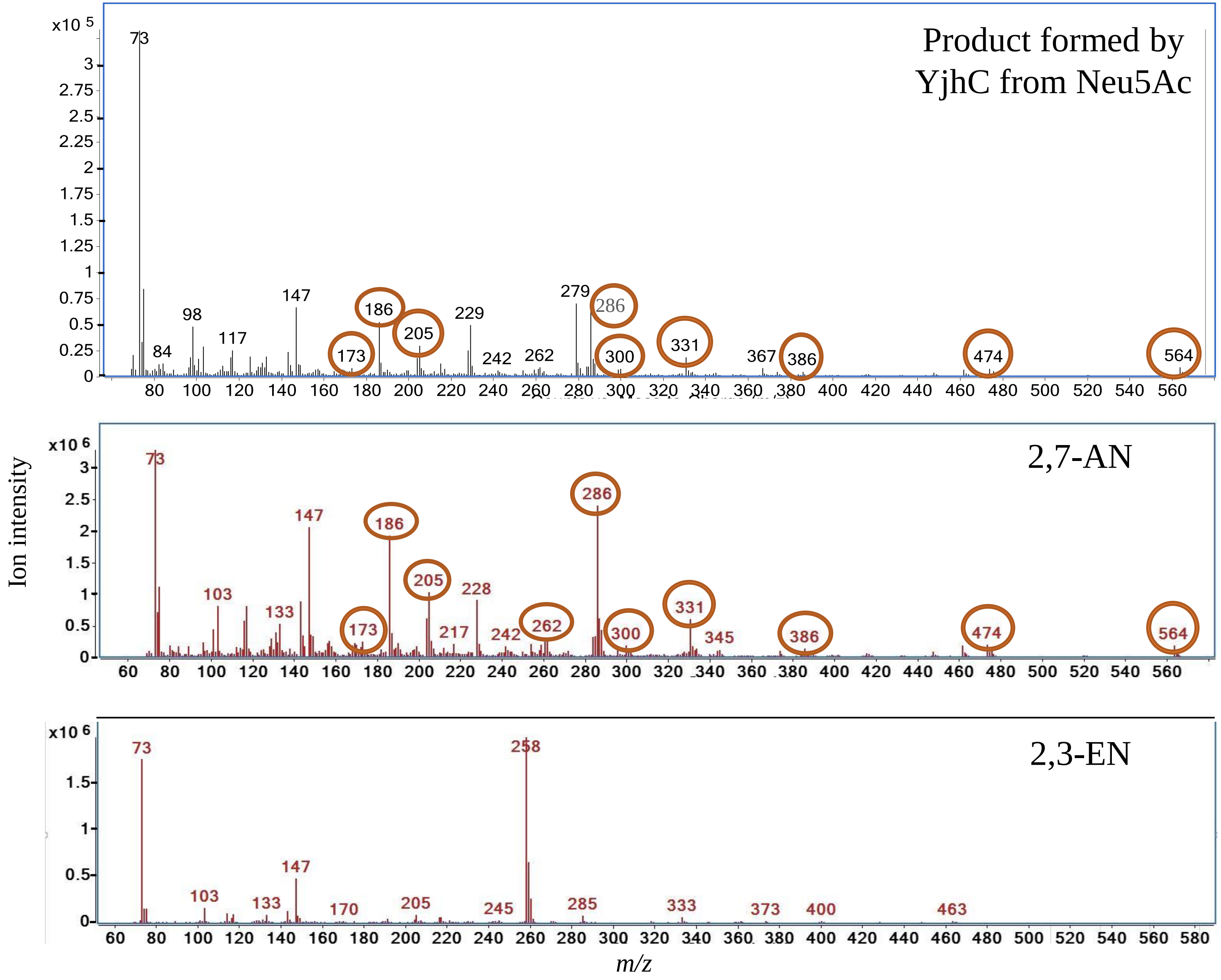
Product formed by YjhC from Neu5Ac
2,7-AN
2,3-EN
Ion intensity
m/z

FIGURE S1

Figure S1: GC-MS analysis of the dehydrated product formed from Neu5Ac by YjhC. The spectrum of the TMS derivative of the product formed from Neu5Ac by YjhC is compared to those of the silyl derivatives of 2,7-AN and 2,3-EN obtained by analysis of 2,7-AN and 2,3-EN standards. The spectrum of the product shows that 10 peaks are in common with those of the 2,7-AN (in agreement with Suzuki *et al*., 1984). This spectrum is different from the one of 2,3-EN.

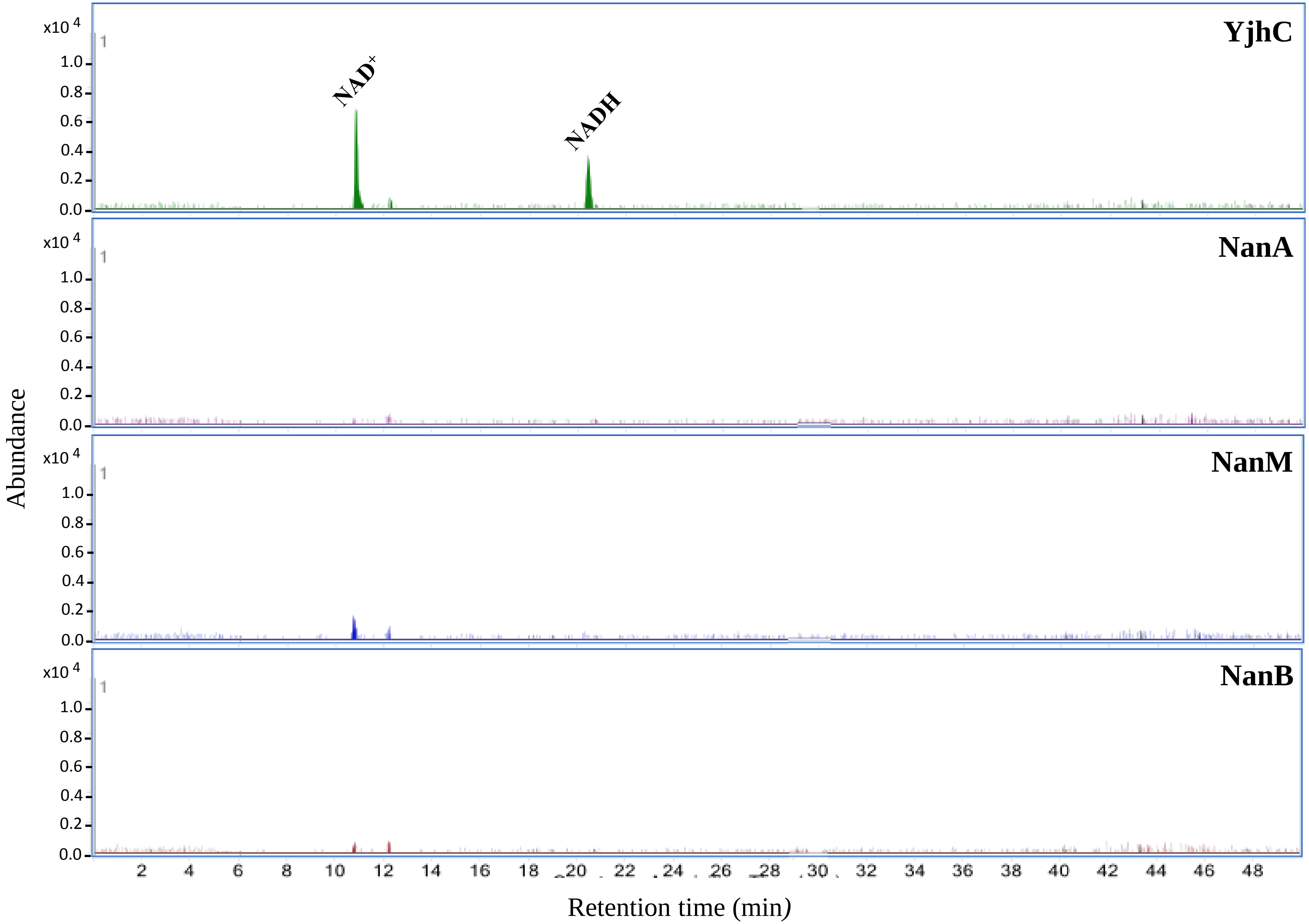

YjhC
NanA
NanM
NanB
NAD+
NADH
x10 4
1.0
0.8
0.6
0.4
0.2
0.0
Abundance
2
4
6
8
10
12
14
16
18
20
22
24
26
28
30
32
34
36
38
40
42
44
46
48
Retention time (min)

FIGURE S2

<u>Figure S2:</u> Presence of NAD^+ and NADH in the preparation of purified YjhC. The figure shows extracted ion currentsfor NAD^+ (*m/z* 662.1014) and NADH (*m/z* 664.1180) in LC-MS chromatograms on which samples of denatured (purified) recombinant proteins. The following proteins were analysed: *E. coli*YjhC (1.5 nmol), NanA (1.5 nmol) and NanM (1.5 nmol), and *S. pneumoniae* sialidase B (0.8 nmol). The identity of the NAD^+ and NADH peaks was ascertained by the exact *m/z* value and by coelution with standards. From these data we calculated that the preparation of YjhC contained 0.094 mol NAD^+ and 0.008 mol NADH/mol of protein, respectively.

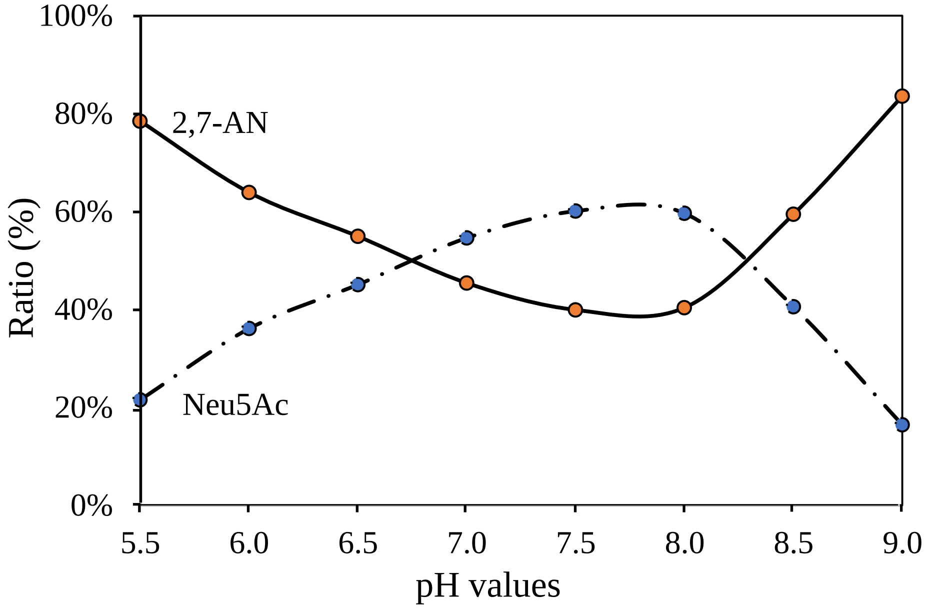

100%
80%
60%
40%
20%
0%
Ratio (%)
2,7-AN
Neu5Ac
5.5
6.0
6.5
7.0
7.5
8.0
8.5
9.0
pH values

FIGURE S3

<u>Figure S3:</u> Effect of pH on the 2,7-AN hydrolase activity of YjhC.

The assay was performed in two steps (as in Fig. 3). The incubation mixture contained the following buffers (50 mM) : MES (pH-5.5-6.5), HEPES (pH 7-8), Tris (8.5-9), as well as 10 mMKCl, 1 mM DTT, 1 mM $MgCl_2$, 50 mM NAD^+, 0.5 mg/mL bovine serum albumin, 0.45 µM YjhC and 1 mM 2,7-AN. The assay was performed in a volume of 200 µL and at 37 °C. After 15 min incubation, the reactions were arrested by heating at 80 °C for 5 min. Concentrations of residual substrates and formed Neu5Ac were measured spectrophotometrically.

A

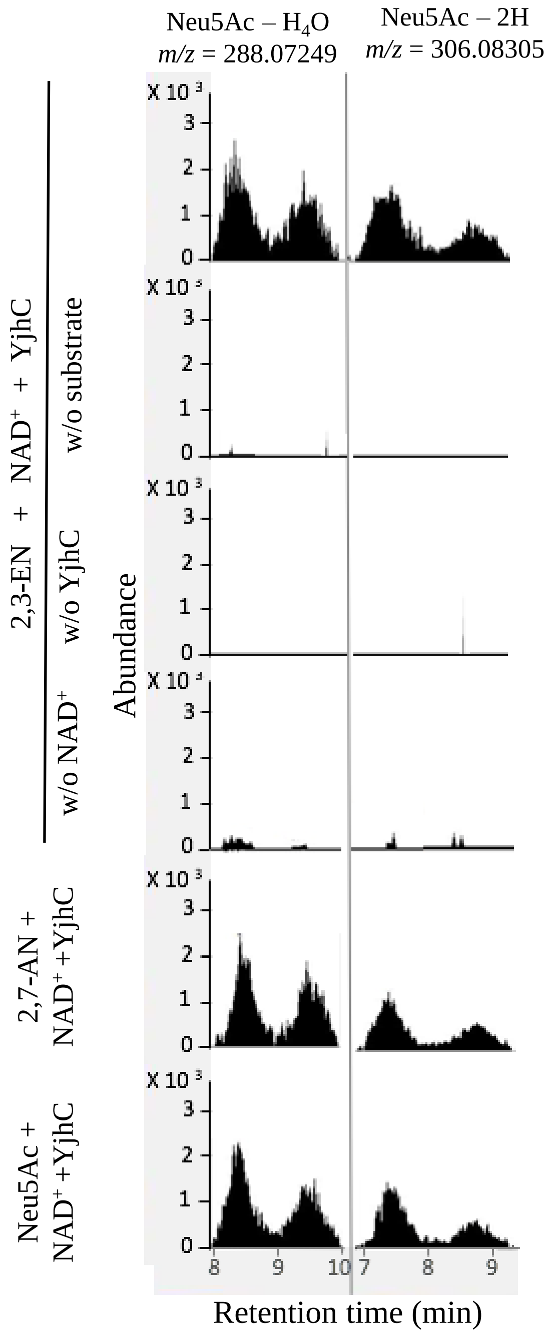

Neu5Ac – H4O
m/z = 288.07249
Neu5Ac – 2H
m/z = 306.08305
2,3-EN + NAD+ + YjhC
w/o substrate
w/o YjhC
w/o NAD+
2,7-AN + NAD+ +YjhC
Neu5Ac + NAD+ +YjhC
Abundance
X 10 3
3
2
1
0
8
9
10
7
8
9
Retention time (min)

FIGURE S4

Figure S4: Formation of dehydro (-2H) and dehydro-anhydro-(-H_4O) derivatives of sialic acid in the presence of substrate and NAD^+. LC-MS analysis showing the appearance of peaks corresponding to putative intermediates in a reaction mixture containing, as indicated, 1 mM 2,3-EN, 2,7-AN or Neu5Ac, 0.45 µM YjhC and 30 µM NAD^+.

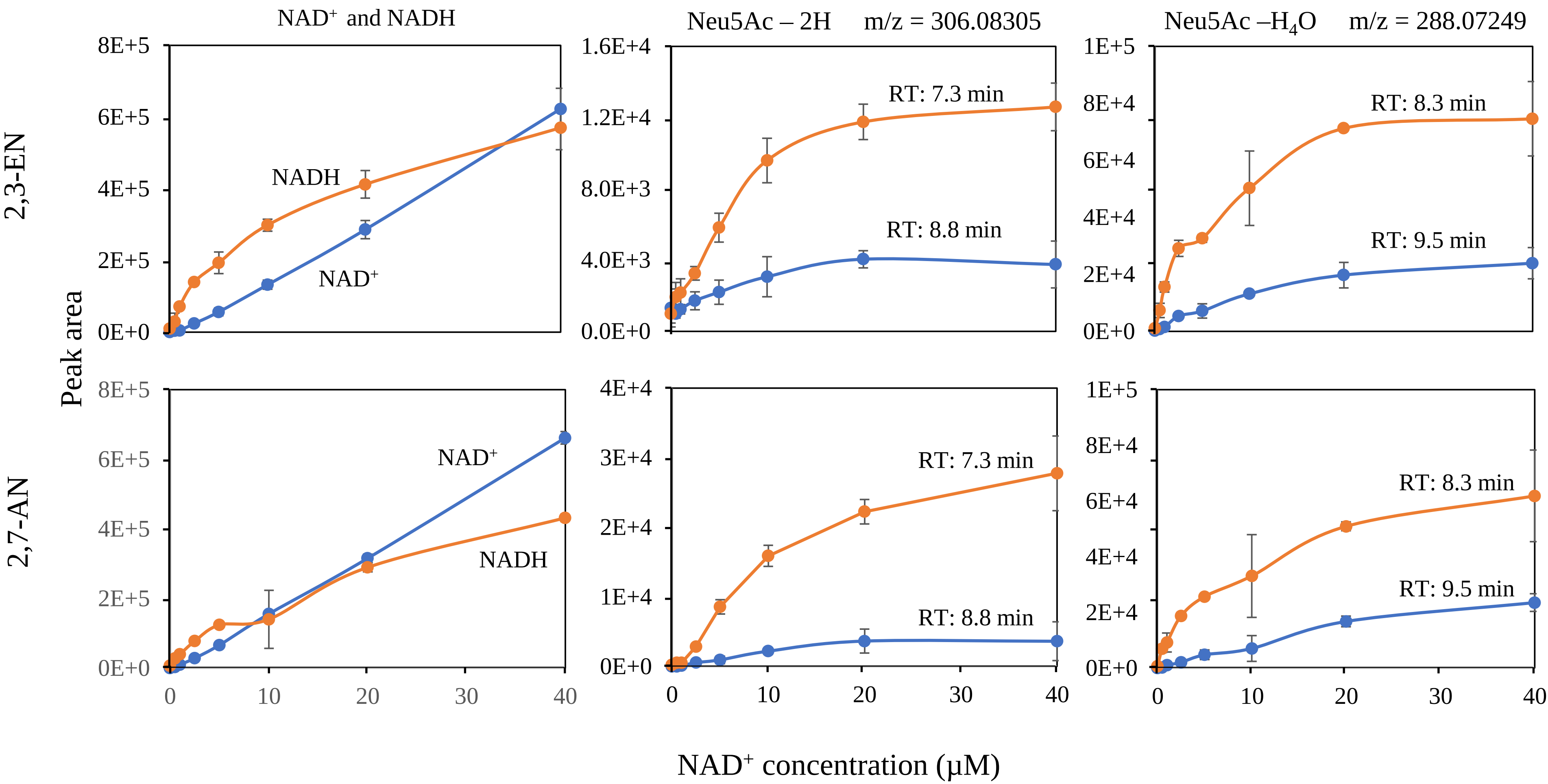
NAD+ and NADH
Neu5Ac – 2H m/z = 306.08305
Neu5Ac –H4O m/z = 288.07249
2,3-EN
2,7-AN
Peak area
NADH
NAD+
RT: 7.3 min
RT: 8.8 min
RT: 8.3 min
RT: 9.5 min
NAD+ concentration (µM)

FIGURE S5

Figure S5: **Effect of NAD^+ concentration on the formation of NADH and intermediates.**

Same experiment as in Fig. 4.

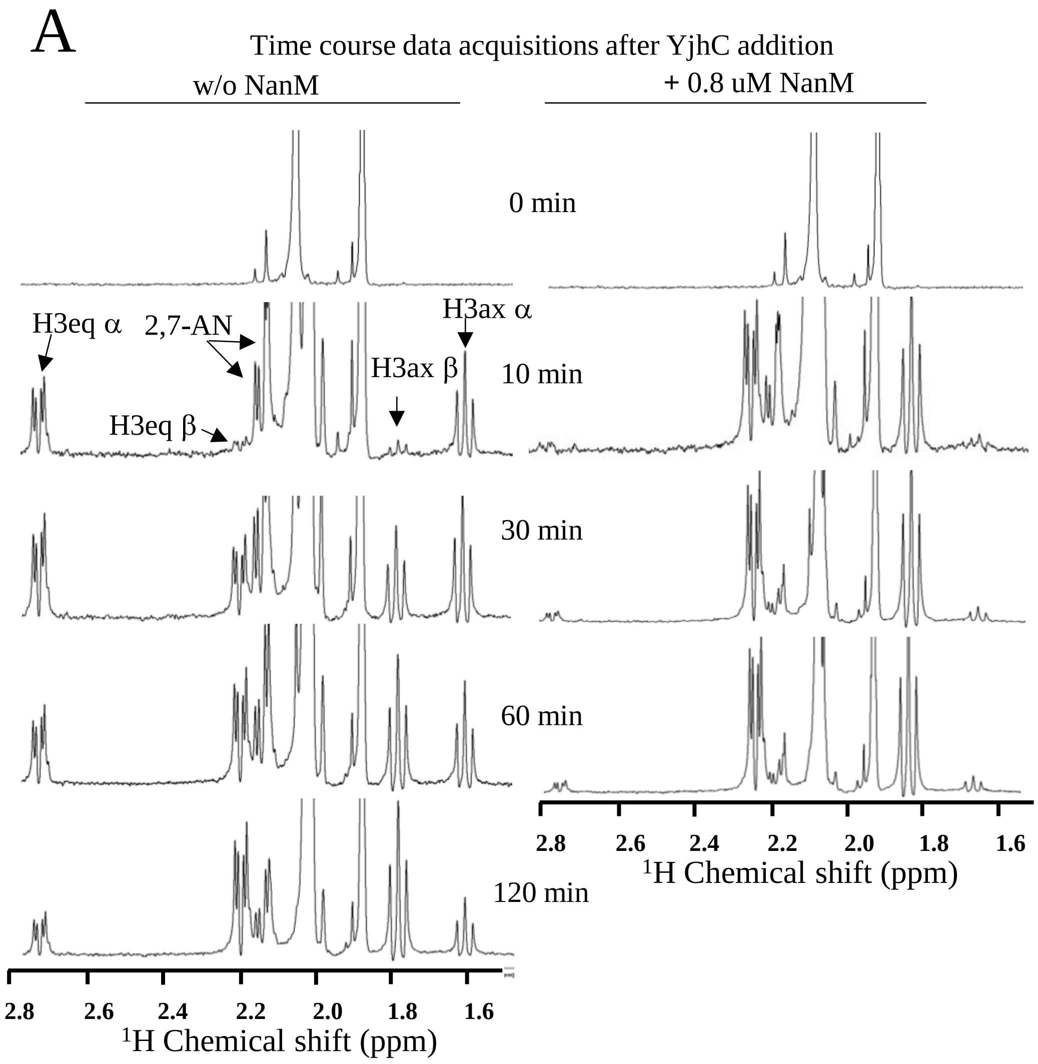
A
Time course data acquisitions after YjhC addition
w/o NanM
+ 0.8 uM NanM
0 min
10 min
30 min
60 min
120 min
H3eq α
2,7-AN
H3eq β
H3ax β
H3ax α
2.8
2.6
2.4
2.2
2.0
1.8
1.6
¹H Chemical shift (ppm)

FIGURE S6

B

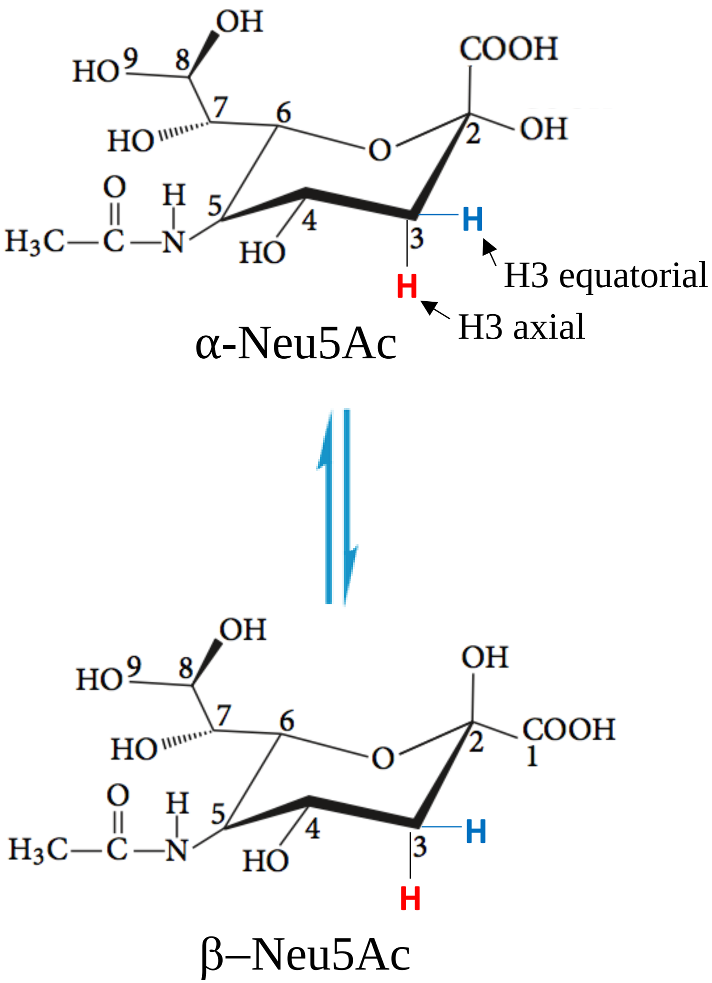

C: ¹H NMR standards

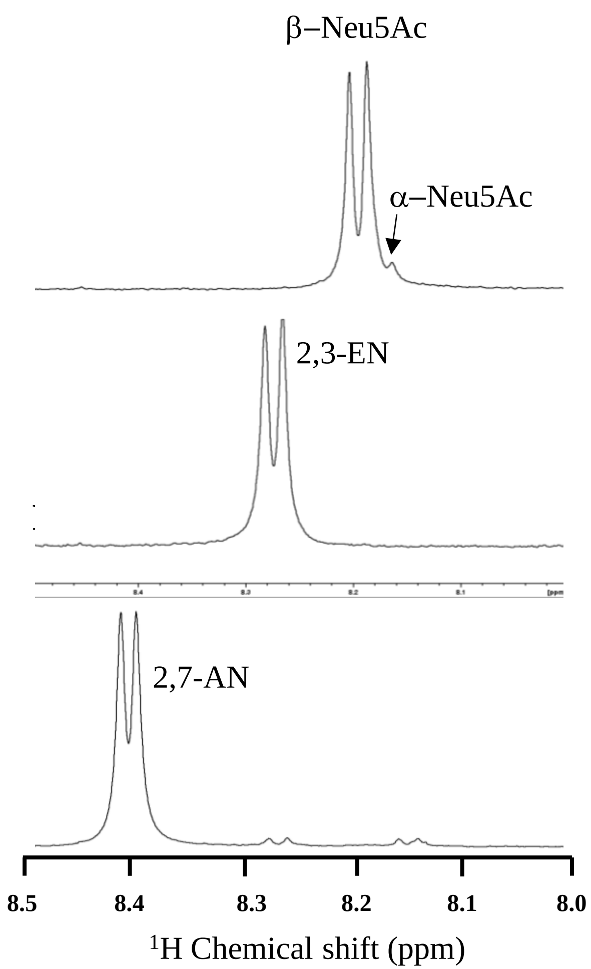

FIGURE S6

<u>Figure S6:</u>NMR study of the reaction of YjhC with 2,3-EN.

A:Same experiment as in Fig. 7. The figure shows the 1.6-2.8 ppm region for the indicated scans. The shifts corresponding to axial and equatorial H3 are indicated. B: Structure of the alpha and beta-anomer of Neu5Ac. C: NMR spectra of Neu5ac, 2,3-EN and 2,7-AN showing the 8 ppm region comprising the shifts of N5-H. These peaks were used to quantify the three compounds.

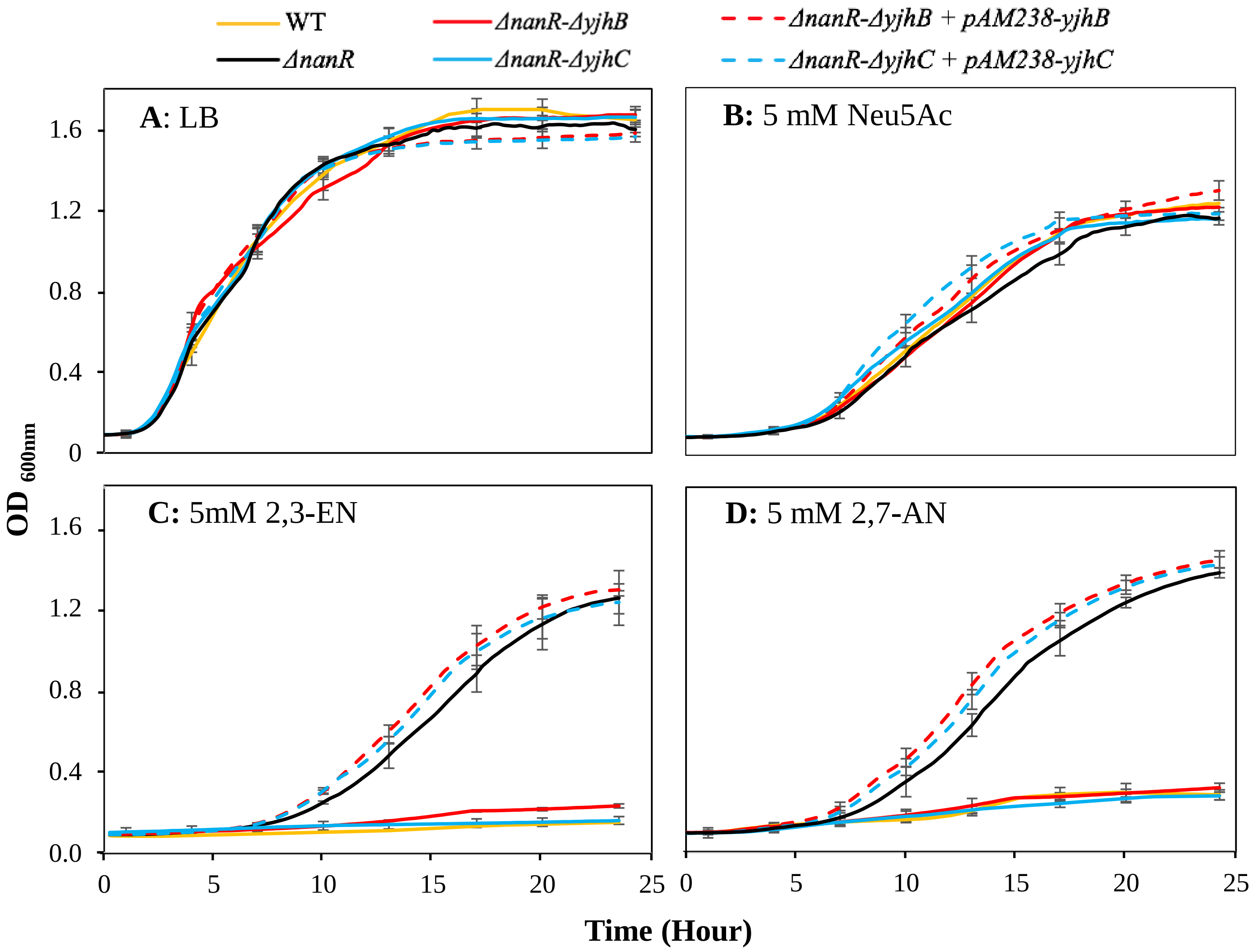
WT
ΔnanR
ΔnanR-ΔyjhB
ΔnanR-ΔyjhC
ΔnanR-ΔyjhB + pAM238-yjhB
ΔnanR-ΔyjhC + pAM238-yjhC
A: LB
B: 5 mM Neu5Ac
C: 5mM 2,3-EN
D: 5 mM 2,7-AN
OD 600nm
Time (Hour)
0
0.0
0.4
0.8
1.2
1.6
0
5
10
15
20
25

FIGURE S7

<u>Figure S7:</u> Growth of *E. coli* on 2,3-EN or 2,7-AN depends on the inactivation of NanR and on functional YjhC and YjhB.

Growth of WT, Δ*nanR*, Δ*nanR*-Δ*yjhC* and Δ*nanR*-Δ*yjhB* and YjhC or YjhB complemented strains on LB medium (A) or M9 medium containing 5 mM Neu5Ac (B), 2,3-EN (C) or 2,7-AN (D).
